# Developing a sustainable grease from jojoba oil with plant waste based nanoadditives for enhancement of rolling bearing performance

**DOI:** 10.1038/s41598-023-50003-9

**Published:** 2024-01-04

**Authors:** Ndabezinhle Ngubhe Dube, Marwa ElKady, Hussien Noby, Mohamed G. A. Nassef

**Affiliations:** 1 Chemical and Petrochemicals Engineering, Egypt-Japan University of Science and Technology, New Borg El-Arab City, 21934 Egypt; 2 Fabrication Technology Department, Advanced Technology and New Materials and Research Institute (ATNMRI), The City of Scientific Research and Technological Applications, Alexandria, Egypt; 3 Materials Engineering and Design, Faculty of Energy Engineering, Aswan University, Aswân, 81528 Egypt; 4 Industrial and Manufacturing Engineering Department, Egypt-Japan University of Science and Technology, New Borg El-Arab City, 21934 Egypt; 5 Production Engineering Department, Alexandria University, Alexandria, 21544 Egypt

**Keywords:** Mechanical engineering, Nanoscale materials, Nanoscale materials

## Abstract

This paper presents a novel grease from jojoba oil and activated carbon nanoparticles (ACNPs) extracted from banana peel waste. The raw jojoba oil and ACNPs are first characterized for structural properties. Samples of jojoba grease blended with 0.5 and 1.5 wt. % ACNPs are prepared and tested for physicochemical and tribological properties as compared to plain jojoba grease. Adding ACNPs to jojoba grease improves corrosion resistance from grade 2c to 1a while increasing the dropping point from 100 to 109 °C. ACNPs enhanced the viscosity of jojoba oil by up to 33% for testing temperature range of 40–100 °C. The load-carrying capacity of jojoba grease is increased by about 60% when blended with 1.5 wt.% ACNPs. The same blending decreased both the coefficient of friction and the wear scar diameter by 38% and 24%, respectively. A customized test rig is used to test the effectiveness of the grease samples in rolling bearing lubrication in terms of vibration levels and power consumption. The novel jojoba grease proved to show exceptional reductions power consumption reaching 25%. The vibration spectra show the absence of resonant peaks at high frequencies suggesting the capability of jojoba grease to form a stable full film lubrication.

## Introduction

Lubricants are essential petroleum consumables in the contemporary world that are used to reduce friction, wear, temperature, and vibrations in operating mechanical systems^1,2^. As global energy consumption is expected to increase to more than 700 Exajoules in 2040 from 584 Exajoules in 2019, a corresponding growing demand for oil and grease lubricants will be needed in different sectors, such as power plants, transportation systems, gas and petroleum plants, manufacturing lines, and paper mills^3^.

Grease is a semi-solid lubricant made by dispersing a thickening agent in a liquid oil^4–8^. In comparison to lubricating oils, a typical grease structure consists of (5–35%) thickening agent (usually metal or polymer soap), (65–95%) base oil (mineral, synthetic, or esters), and (0–10%) additives. Usually, additives are added in small quantities to enhance or introduce particular properties such as corrosion resistance, oxidation stability, extreme pressure enhancement, and lubricity improvement^4,9^.

Most lubricating greases are made from mineral oils, which are not only non-renewable but also harmful to the environment during their extraction, processing, and disposal phases^2^. Therefore, there is an increasing interest in exploring alternatives that are more sustainable and biodegradable. Vegetable oils have been proposed by many researchers as substitute lubricants to mineral oils because of their superior properties, such as lower evaporation rates, higher viscosity index, biodegradability, lower toxicity, and better lubricity^10,11^. Despite these advantages, vegetable oils suffer from poor thermal and oxidative stabilities^12^. In addition, the effectiveness of vegetable oils as lubricants has been found to decline when they are subjected to increasing extreme loads compared with mineral oil lubricants^13^.

Furthermore, vegetable oils can be more corrosive than mineral oils when used for lubricating machinery components in the presence of moisture, the absence of suitable corrosion inhibitors, elevated temperatures, and dissolved oxygen^12,14,15^. This is mainly attributed to their higher affinity for water in addition to the existence of unsaturated fatty acids. As a consequence, this would degrade the equipment’s performance over time. Jayadas and Nair explained the important mechanisms of corrosion induced when applying vegetable oils as industrial lubricants^16^. The moisture content in vegetable oils was identified as the leading cause of corrosion in vegetable oil-based lubricants.

The properties of vegetable oils can be improved by using lubricant additives, including corrosion inhibitors, anti-wear agents, friction modifiers, and extreme pressure agents^17^. However, commonly used additives contain elements such as sulfur and phosphorus, which have been proved to cause environmental pollution^18,19^. For example, Zinc dialkyldithiophosphate (ZDDP) is a widely used lubricant additive in engines due to its excellent anti-oxidation properties. However, ZDDP can cause catalyst poisoning and electrolytic corrosion, which directed many researchers to find other alternatives as lubricant additives^20^. Furthermore, most of these additives are found effective for mineral oil lubricants while showing poor performance with vegetable oils^21^.

Toxic and corrosive slurries are widely employed in chemical mechanical polishing (CMP), resulting in pollution to the environment. To overcome this challenge, nanoparticles are used to develop novel green CMP for copper^22^, sapphire^23^, alloys^24^, diamond^25^, silicon^26^ and fused silica^27^. Using the developed green CMP, high-performance surfaces are achieved for the use in semiconductor, aerospace and optoelectronics industries^28^. The most important is that these studies are a great contribution to the conventional CMP and manufacturing^29^. Moreover, a novel approach of single grain sliding is developed at 40.2 m/s and nanoscale depth of cut^30^, and stress, depth of cut, force and size of plastic deformation are proposed^31^. This method opens a new pathway to investigate the fundamental mechanisms of wear and friction^32^.

Nanoparticle additives are currently emerging as a novel alternative to enhance the properties of vegetable oils. Kulkarni et al.^33^ studied CaCO_3_ nanoparticles as an environmentally friendly additive for jojoba oil. The study reported a decrease in wear scar diameter (WSD) and the coefficient of friction (COF) by as much as 40.2% and 34.1%, respectively, when adding 0.3 wt. % CaCO_3_. Another investigation by Arumugam et al.^34^ showed that the addition of 0.05 wt. % TiO_2_ nanoparticles to modified rapeseed oil reduced the friction coefficient and the wear scar diameter by 15.2% and 11%, respectively. Other studies focused on studying the influence of nanoparticles on generated mechanisms of friction, wear, and plastic deformation during manufacturing processes^31,35^. For instance, a novel approach is conducted by sliding a nanosized diamond grit on a single silicon crystal at nanoscale depth of cut with a speed of 40 m/s. The corresponding stress, force and size of plastic deformation were calculated^30^. Traditional polishing slurries usually contain toxic and corrosive ingredients, resulting in pollution to the environment^25^. To solve this problem, novel green slurries were developed for use in semiconductor industries^29^.

Many current research works have explored the effect of applying nano additives to vegetable oils^18,36–38^, but comparatively little work was reported on bio-greases^39,40^. Nagendramma et al.^9^ used lithium soap and ZDDP antiwear additives to prepare a bio-grease from residual vegetable oil. The dropping points of the proposed grease reached 195 °C. Studies with carbon-based nano-additives have shown that their incorporation into greases improves their physical and mechanical properties^41–44^.

The previous outcomes have inspired researchers to further explore the effect of nanoadditive materials on jojoba oil as one of the promising inedible vegetable oils. Jojoba oil is an unsaturated liquid wax consisting of nearly 98% wax esters^45^. The fatty acid structure of jojoba oil consists of a high content of monounsaturated eicosanoic acid with traces of oleic acid^46^. It possesses high lubrication qualities with satisfactory stability in the temperature range of 40–100 °C^47^. When reinforced with graphene nanoadditives, modified jojoba oil with SAE20 W40 showed a significant reduction in COF values^48^. Another work studied the effect of dispersion of Al_2_O_3_ nanoparticles in jojoba oil on its tribological performance using a pin-on-disk tribosystem^49^. The addition of 0.2 wt.% alumina concentration reduced the COF and WSD by 30% and 21%, respectively.

From the review of the literature, jojoba oil is unique in structure and different from other vegetable oils. It is characterized by a high viscosity index and high thermal oxidative stability due to its long-chain monounsaturated fatty ester structure. It is also obtained from a sustainable plant that is drought resistant. Due to its distinctive properties, much more investigations should be conducted on jojoba oil as a futuristic base oil in industrial lubricants^39^. This paper presents a novel eco-friendly grease from unmodified jojoba oil as a base oil and lithium stearate as a thickener. To enhance its physicochemical and tribological performance for machinery lubrication, activated carbon nanoparticles (ACNPs) are fabricated in this work and applied at different concentrations to the developed bio-grease samples. In previous works by the authors^42^, ACNPs was applied to lithium grease at 0.025 wt.%, 0.05 wt.%, 0.1 wt.%, 0.5 wt.%, and 1 wt.% blends. All five blends were found to be effective in reducing COF and WSD while enhancing the load carrying capacity of grease. The blend of 0.5 wt.% ACNP showed the best tribological results as individual nanoadditive and as a hybrid additive with reduced graphene oxide. Therefore, 0.5 wt.% ACNPs was selected in this work for physicochemical characterization and tribological testing to evaluate the performance of ACNP obtained from banana peel waste in lithium grease. Also, 1.5 wt.% ACNPs is selected in this work to evaluate the effect of higher concentrations of this nanoadditive on physicochemical, tribological and dynamic performance and whether it would cause undesired agglomeration. Raw jojoba oil was purchased and characterized for its physical and chemical properties. Then, bio-grease samples are prepared and tested for corrosiveness tendency and consistency at elevated temperatures in the absence and presence of ACNPs using the copper corrosion test (ASTM D130) and dropping point test (ASTM D2265). The tribological performance of the developed bio-grease samples are compared to that of commercial lithium grease using the Brugger test setup to determine the average WSD, load-carrying capacity, and average COF. A special test setup is used to test and validate the performance of the developed grease on rolling bearings in terms of vibrations levels and power consumption.

## Materials and methods

### Preparation and characterization of ACNPs

Activated carbon (AC) is prepared in this work according to the methods described by Gohoho et al.^50^. With an estimated 1,285,129.25 tons of bananas annually consumed in Egypt, banana peel waste is selected in this work as a sustainable and economic raw material for AC production. Furthermore, the preparation procedure of the ACNPs from the banana peels is rather simple which promote AC to be an economic carbonaceous nanoadditive when used in commercial lithium grease in comparison with considering other expensive similar alternatives such as reduced graphene oxide (rGO) and multi-wall carbon nanotubes (MWCNTs)^51^. Banana peels are first collected and thoroughly washed with distilled water. The peels are then chopped into small pieces and sun-dried for 4 days. They are further oven dried at 100 °C for 24 h to eliminate all moisture. The dried peels are then carbonized at 500 °C for 2 h in a nitrogen atmosphere using a gas furnace (GCF 1600 Gas Furnace Across International) with a heating rate of 5 °C/min. The carbonized peels are impregnated with 1 M solution of KOH at a 1:1 mass ratio and then stirred for 2 h using a magnetic stirrer. The mixture is allowed to stand for 24 h before being filtered and rinsed with distilled water and HCl to achieve a neutral pH. The washed peels are carbonized again for 2 h at 500 °C to produce activated carbon (AC), which is milled in a planetary ball mill (PM 400, MA Type, Retsch) to reach nanosized particles of AC powder.

Scanning electron microscopy (SEM) (JEOL, JSM-6010LV) is used to investigate the ACNPs microstructure. A Shimadzu 6100 diffractometer is used to determine the X-ray diffraction patterns of the prepared AC. The functional groups of the prepared AC are identified using Fourier transform infrared (FTIR) spectrometer with a wavelength range of 400–4000/cm (Bruker Vertex 70, Bruker Company, Billerica, MA, USA).

### Preparation of jojoba grease

Jojoba crops are favorably planted and cultivated in dry and semi-desert territories such as Egypt^52^. In this work, jojoba oil is purchased from the local market in the crude form of a yellow liquid wax. Three sets of grease samples (G1, G2, and G3) are prepared in batches using an open kettle with a magnetic stirrer. Jojoba oil (80%) is used as the base oil with lithium stearate (20%) as a thickener and ACNPs as additives. G1 sample is jojoba grease without ACNPs (0 wt.% ACNPs), G2 is Jojoba grease blended with 0.5 wt.% ACNPs, and G3 is Jojoba grease blended with 1.5 wt.% ACNPs.

First, 60% of the base oil is heated to 110 °C for 10 min to remove moisture. The stearic acid is added to the oil and the mixture is stirred until the acid dissolved in the oil. The temperature is raised to 160–180 °C and aqueous lithium hydroxide is added dropwise for the saponification stage. The mixture is stirred at this temperature for 1 h to allow complete saponification between the stearic acid and the lithium hydroxide to occur before forming lithium stearate soap thickener. When saponification is completed, the temperature is controlled to drop to 100 °C and stirring is continued for another 1 h. During this stage, ACNPs additives and the rest of the base oil are added to the mixture. By the end of the specified duration, the heat source is turned off, and the mixture is stirred for another 30 min before it is left to cool at room temperature. A commercial synthetic lithium grease is purchased from a local supplier for comparison. The main constituents of the base oil in lithium grease are assessed using FTIR spectrometer and physicochemical analysis.

### Viscosity and density tests

The kinematic viscosity of jojoba oil is tested in its plain format and also with the ACNPs additions (0.5 wt.% and 1.5 wt.%) at 40, 60, 80, and 100 °C, according to ASTM-D445. The purpose of this test is to identify the optimum operating temperature for jojoba oil as a lubricant in machinery. Also, the test is essential to investigate the effect of the carbonaceous nanoadditives on the kinematic viscosity values. During the test, a 30 mL sample is set to flow through a capillary viscometer in a liquid bath at the preset temperature. Another indicator that is assessed in this work is the viscosity index. A higher viscosity index indicates that the fluid's viscosity changes less with temperature, making it more stable and less prone to thickening or thinning under different conditions. The density of crude oil samples is tested using a density meter at 15 °C using ASTM 4052.

### Pour point test

The pour point is an appropriate indicator for the low-temperature properties of oil. The pour point is a vital characteristic of lubricant quality for machinery operating in low-temperature areas. According to ASTM-D97-17b, this property is evaluated by adding a 30 mL oil sample to a test jar that has been heated to a temperature of 45 °C. A higher-range thermometer is used to measure the pour point value after the jar has been allowed to cool in a cold bath. The difference between the pour point and the temperature at which oil no longer flows is 3 °C in a horizontally fixed sample container.

### Acid value test

The acid value of an oil is a measure of the breakdown of triacylglycerols into free fatty acids ^53^. It is determined by the number of milligrams of potassium hydroxide required to neutralize the free acid in 1 g of the substance. A low acid value is desirable in good oil, as it indicates a low level of oxidation. An increase in acid value can be an indicator of oil oxidation, which may lead to gum and sludge formation as well as corrosion^54^. The acid value of a vegetable oil is determined using a pH-metric method without titration. The oil sample is mixed with a reagent containing triethanolamine, isopropanol, and water. Then, the pH of the mixture is measured. The acid value is calculated from the difference between the initial pH and the final pH after the addition of a standard acid.

### Copper corrosion test

To determine the resistance of the developed jojoba grease to corrosion, a copper corrosion test is conducted on the grease samples according to the standard test method ASTM D130. Another purpose of the test is to confirm the absence of harmful sulfur element in grease. A strip of polished copper is fully submerged in the test grease sample and then heated to 100 °C ± 1 °C for 24 h ± 5 min. By the end of the heating period, the strip is removed from the sample and washed with acetone. The ASTM Copper Strip Corrosion Standard is then used to compare the tarnish and color level of the strip to determine its rating.

### Dropping point test

The dropping point of the grease samples is determined using ASTM D2265. This test is essential to determine the cohesiveness of the grease during service in machinery structure at elevated temperatures. The dropping point temperature is an indicator of the beginning of the weakening of the grease fibrous structure leading to base oil bleeding from the grease. A grease sample inside a test cup is heated at a specified temperature inside an aluminum block oven. A thermometer is used to measure the temperature in the oven without making contact with the grease sample. When the first drop of grease oil fell from the cup, the temperature is recorded, whereas the oven temperature is simultaneously noted. The two values are used together with a correction factor to obtain the dropping point of the grease.

### Brugger test

The load carrying capacity for the grease samples is determined using a customized tribometer. The tribometer is developed based on Brugger’s test apparatus (DIN 51347-1), which uses a roller against a ring to determine the tribological properties of the lubricant. The modified tribometer contains a lever mechanism with test weights on one end and a cavity with roller elements on the other end with 8 g of grease. The roller elements are positioned to rotate at a 90° angle against a spinning ring. In the operation of the tribometer, the motor is first run for 30 s to allow uniform distribution of grease. Thereafter, a 5 N load is attached to the lever, and the tribometer is run for 10 min ± 15 s at 800 ± 5 rpm. If welding does not occur within the time period, the load is removed, and roller elements are examined for surface wear. An additional 5 N load is then added, and a new roller element is used to run the tribometer under the same conditions again. Loads are successively added, and tests are carried out until welding occurs.

### 4-Ball wear test

The COF and WSD are determined using a 4-ball wear test apparatus according to ASTM D5183 and ASTM D2266, respectively. The tester contains a rotating ball atop three stationary balls in a cup filled with the test lubricant. During the test run, the lower rotating balls revolve at 1200 ± 60 rpm, and the whole system operates at 75 ± 2 °C with a 40 kg load pressing the top ball for 60 ± 1 min. After the operation, the test balls are analyzed for wear using a microscope to determine the wear scar diameter. To determine the COF, successive 10 kg loads are added at 10 min intervals up to the point of welding/seizure between mating surfaces. Each test is repeated three times, and the average and standard deviation are determined.

### Bearing test-setup

**Figure 1:**
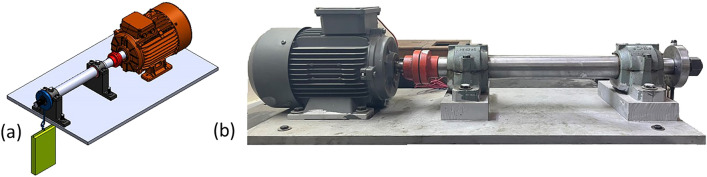
(**a**) CAD model of rolling bearing and (**b**) experimental test setup^30^.

A specially designed and constructed test setup is used in this work to conduct running tests on rolling bearings lubricated with the novel grease, as shown in Fig. 1. The test rig consists of an induction motor, a flexible coupling, a base, a rotating shaft, two supporting bearings, and a test bearing with the applied radial load. The main design specifications of the test setup are described in detail by Nassef et al.^43^.

The selected test bearing is NSK 6006zz deep groove ball bearing^43^. The test bearing is fitted to its ring-shaped housing, which is snug fitted to the rotor. The motor is set to run at 1400 RPM under two different radial loads of 100 N and 200 N applied to the test bearing. The selected loads correspond roughly to around 1% of the bearing dynamic load carrying capacity.

The performance of each grease blend is evaluated using vibration measurements and power consumption levels during each running test under the two different radial loads. The vibrations signals are measured using an accelerometer attached to the test bearing housing in the radial vertical direction. The signals are then recorded, conditioned, and processed using Commtest VB 5 vibration analyzer according to ISO 10816 part 7. Each vibration is measured as velocity amplitudes (mm/s). Root Mean Square (RMS) is selected as an indicator for the signal vibration amplitude in time domain and frequency domain because it is directly proportional to the consumed energy in bearings.

## Results and discussion

### Characterization of developed ACNPs from banana peels

**Figure 2:**
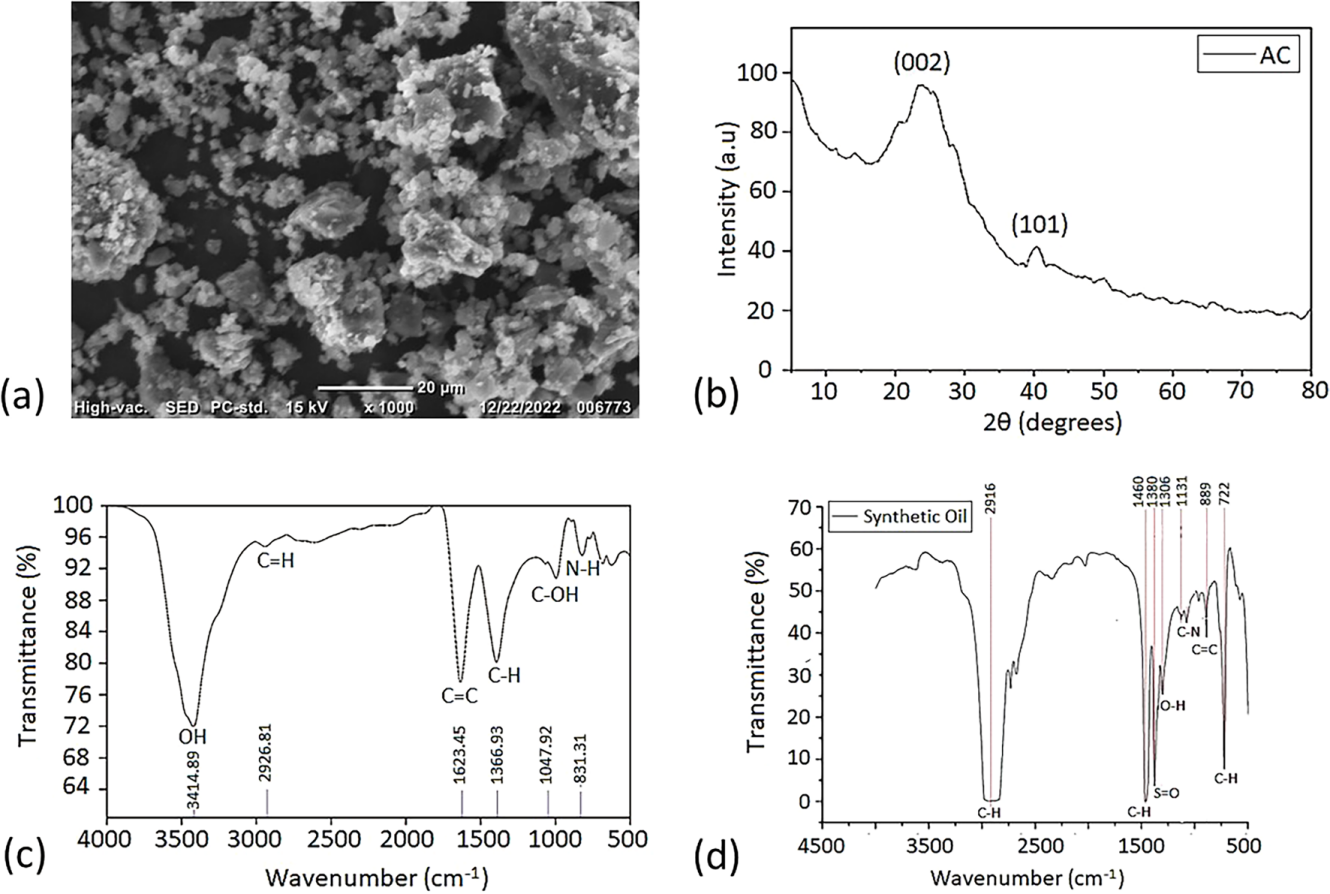
(**a**) SEM of ACNPs, (**b**) XRD patterns of the*,* (**c**) FTIR spectra of the AC nanostructure and (**d**) FTIR spectrum of synthetic base oil in commercial lithium grease.

Figure 2a shows the SEM results of the prepared AC nano-powder. The surface morphology shows aggregated particles of bulky compact shapes with varying sizes indicating a continuous consumption of material due to the activation of the interior and exterior of AC. The range of ACNP sizes is between 20 and 55 nm with an average value of approximately 37 nm. Figure 2b shows the X-Ray diffraction analysis (XRD) patterns of the prepared ACNPs. There is a broad peak at 2θ ≈ 24° and a weaker peak at 2θ ≈ 42°. The broad peak represents the (002) carbon plane (JCPDS card no. 87-1526) and confirms that the prepared AC is largely amorphous. The weaker peak corresponds to the (101) phase reflection for highly graphite-like carbons^55^.

Figure 2c shows the Fourier transform infrared (FTIR) spectra of the prepared AC at wavelength range between 500 and 4000/cm. The first significant peak in the FTIR spectrum is a broad peak at 3414/cm corresponding to phenolic group OH stretching, which shows the presence of free hydroxyl groups (the presence of water) in the developed nano-activated carbon. Another significant peak exists at 2926.81/cm, which corresponds to the C=H stretching of alkenes and aldehydes. The peak at 1623.45/cm is attributed to the C=C stretch of aromatic rings, which also indicates an acceptable level of structural integrity^56^. The peak at approximately 1387/cm corresponds to the C–H stretching in CH_3,_ which shows the strong functionalization of hydrogen with carbon atoms. The peaks at 1048/cm and 831/cm correspond to C–OH and N–H amine groups, respectively.

### Physicochemical properties results

The chemical composition of the jojoba oil product in terms of fatty acids and sterols is obtained from the supplier using gas chromatography (GC) analysis. GC analysis is conducted using an Ultra GC–MS (Shimadzu, Kyoto, Japan) equipped with an electron impact source. The chemical composition of jojoba oil is compared to previous research works from the literature for validation of values because the geographical location of jojoba plants may have an effect on the output of jojoba oil fatty acid constituents and percentages ^57^. From the supplier data, the main constituents of the tested jojoba oil samples are eicosenoic acid (72.7%), erucic acid (12.5%), and oleic acid (10.6%). This composition is close to those reported in previous investigations^46,58–60^ indicating that jojoba oil composition slightly differs based on the planting origin. The presence of 95% monounsaturated fatty esters along with approximately 5% pentadecanoic acid (C15:0) and palmitic acid (C16:0) in the composition of jojoba oil indicates sufficient oxidation stability levels. In comparison, the FTIR results of the synthetic base oil in commercial lithium grease reveal the presence of C–H, S=O, and O–H function groups, as shown in Fig. 2d. The chemical analysis of the oil confirms the presence of sulfur additive by 14 mg/kg. The intense peak at 2916/cm is characteristic of C–H stretching in alkanes, specifically in the CH2 group, underscoring the aliphatic hydrocarbon backbone prevalent in synthetic oil^61^. Aliphatic C–H bending vibrations are represented by the peaks at 1460/cm and 1380/cm, further reinforcing the presence of alkanes in the molecular structure^62^. The noteworthy and intense peak at 1306/cm is indicative of a sulfur-containing functional group, potentially associated with sulfur dioxide (S=O) stretching vibrations. Concurrently, the peak at 1075/cm suggests the presence of C–O stretching, possibly in ethers or esters. The peak at 889/cm may be related to aliphatic C–H bending or other specific molecular features. Lastly, the peak at 722/cm could be associated with out-of-plane bending vibrations.

Table 1 shows the values of density, kinematic viscosity, viscosity index, wax content, pour point, and copper corrosion of the tested raw jojoba oil. It is noted that jojoba oil has a density of 0.86 g/mL, which is comparable to the base oil in the commercial lithium grease, the jojoba oil in^59^, and the mineral oils in^63^. The kinematic viscosity at 40 °C is 24.72 cSt, which is equivalent to the viscosity grade ISO VG 22 according to the ISO 3448 viscosity classification for industrial lubricants. The kinematic viscosity at 100 °C is 6.56 cSt, which meets the standard margin for this grade. The values are confirmed by previous jojoba oil results from a review of the literature^59,64,65^. Jabal et al^66^ reported that kinematic viscosities of SAE 40 engine mineral oil are 54.15 and 12.14 cSt at 40 and 100 °C, while sunflower oil possessed 46.15 and 10.80 cSt at 40 and 100 °C, respectively. In the present results, jojoba oil shows lower kinematic viscosity by 54% than the SAE40. Still, the presence of long-chain fatty acids such as erucic acids^67^, results in a relatively thick film with viscosity comparable to the commercial lubricant for engine oil SAE10 W, which falls within an acceptable ISO standard grade as a lubricant for industrial machinery^63^. It is therefore concluded that the kinematic viscosity values of jojoba oil are neither too high to lead to oil starvation nor too low to cause weak tribofilm formation and high wear rates.

**Table 1:** Physicochemical properties of raw jojoba oil.

Property	Method	Base oil (Lithium Grease	Jojoba oil (current work)	Sanchez et al.^45^	Mohamed^49^	Chatra et al.^48^
Density, g/mL@15.56 °C	ASTM D-4052	0.87	0.8601	0.862	–	–
Kinematic viscosity (cSt)						
@40° C	ASTM D-445	30.27	24.72	26.6	25	24.92
@100° C		5.79	6.56		6.5	6.43
Viscosity index	ASTM D-2270	137	242.1	–	233	233
Wax content, wt. %	UOP-64	–	7.88	–	–	–
Water content, vol. %	ASTM E-203	< 0.1	0.03	0.03	–	–
Pour point, °C	ASTM D-97	–	12	–	–	1–9
Copper corrosion	ASTM D-130	–	1a	–	–	–
Acid value	Method	–	0.2	0.36	–	0.36

**Figure 3:**
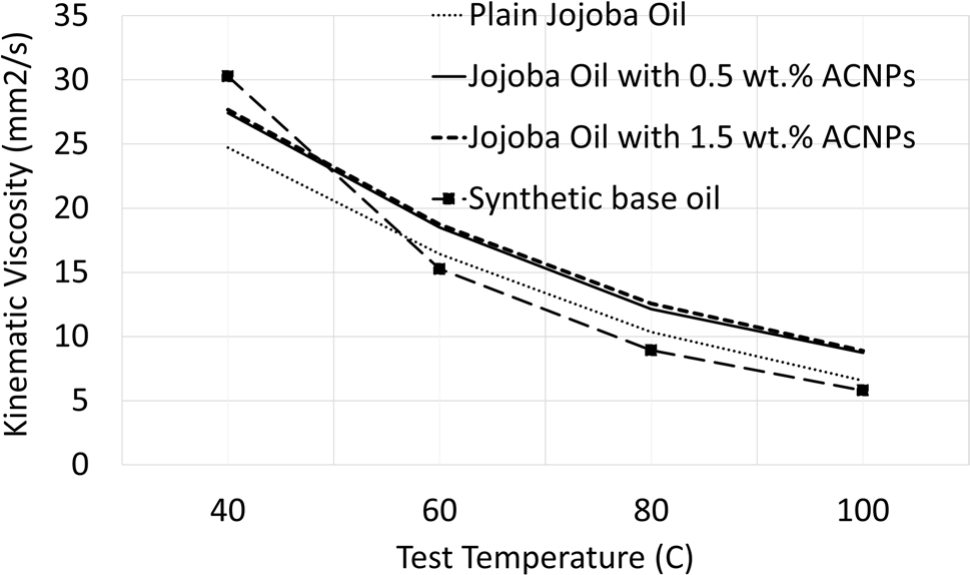
Kinematic viscosity of synthetic base oil and jojoba oil under selected testing temperatures in plain state, with 0.5 wt.% ACNPs, and with 1.5 wt.% ACNPs.

Figure 3 shows the kinematic viscosity with temperature results of jojoba oil in its plain form and with the two concentrations of ACNPs. The results of synthetic base oil in lithium grease are also plotted for comparison. By increasing the temperature from 40 to 100 °C, the kinematic viscosity values fall with an exponential trend. A remarkable enhancement ranging from 11 to 30% in kinematic viscosity is noticed when adding 0.5 wt.% ACNPs to jojoba oil depending on testing temperatures. Increasing the addition of ACNPs to 1.5 wt.% resulted in a slight change in these percentages. The viscosity results of the synthetic base oil in commercial lithium grease are found to be higher by around 20% at 40 °C and fall below plain jojoba oil with the increase of temperature indicating lower viscosity index than jojoba oil.

In Table 1, the value of the viscosity index (VI) for jojoba oil is found to be 242.1, which is high in comparison to synthetic base oil in the commercial lithium grease (137) and other mineral oils which fall in the range between 90 and 100. The VI value is also higher than some vegetable oils (soybean oil VI is 223), indicating excellent stability and resistance to changes in viscosity with temperature^66^. This is particularly advantageous for machinery applications where a consistent viscosity of lubricants is needed; hence, it ensures generating a stable tribofilm over a wider temperature range. The addition of ACNPs in jojoba oil samples increased the VI values to 270 and 280 for 0.5 wt.% and 1.5 wt.%, respectively.

The pour point of jojoba oil is found to be 12 °C, which is much higher than that of commercially used mineral oils (− 9 °C). This is attributed to the dominant amount of saturated fatty esters in jojoba oil and fewer polyunsaturated esters. This issue is generally acceptable in tropical and hot weather regions. On the other hand, these saturated fatty acids and wax esters enhance the oxidation stability of the lubricant, which makes it a competitive candidate for machinery applications where other vegetable oils lack this advantage.

The water concentration % for both jojoba oil and synthetic oil in commercial lithium grease are found to be quite similar (less than 0.1%). The low water concentration % in the case of jojoba oil can be justified by the high hydrophobicity of fatty esters, such as erucic acid^67,68^. This results in low affinity to water and hence a lower possibility for thermal oxidation or turning rancid. Furthermore, the acid value is found to be only 0.2, implying insignificant content of free fatty acids, which agrees with the GC analysis results. The acid value is an important indicator used to evaluate the quality of isolated oil, especially for gears and bearings^69,70^. The copper corrosion rating for raw jojoba oil samples is found to be (1a) due to wax esters that represent a significant portion of its composition^58^. This rating confirms the results of low acid value and low water vol.% reported for jojoba oil.

Table 2 shows the copper corrosion test and dropping point test results for the three jojoba greases samples with and without ACNPs additives. Lithium grease is also tested, in comparison, and is found to show a light tarnish rate (1a), which is justified by the presence of a corrosion inhibition agent in the commercial product. G1 grease sample shows a moderate tarnish of copper strip (2c). This is explained by the fact that lithium soap thickener is lab prepared through the chemical reaction of 1,2-hydroxystearic with lithium hydroxide monohydrate without antiwear additives or anticorrosion agents. The presence of stearic acid in the thickener is expected to chemically react with the copper strip surface and lead to the formation of cupric stearate^71,72^.

**Table 2:** Physicochemical tests for bio-grease samples with and without ACNPs.

Test grease sample	G1	G2	G3	Lithium grease
Copper corrosion test ASTM D 130	2c	1a	1a	1a
Dropping point ASTM D 2265 (°C)	100	106	109	160

It can also be seen from Table 2 that the addition of ACNPs enhances the grease anti-corrosion: grease samples G2 and G3 altered the corrosion rating from 2c to 1a. The AC nanostructure has a high surface area with a vast number of pores and active sites. It can absorb and trap corrosive agents, such as dissolved oxygen, metal ions, and any other aggressive substances present in the surrounding environment. The functional groups on ACNPs can increase their adsorption capacity for corrosive species. For example, polar functional groups such as hydroxyl (–OH), carboxyl (–COOH), or amino (–NH) groups that are identified in the material characterization through FTIR analysis can create additional binding sites for metal ions and other corrosive agents, leading to a more effective adsorption and inhibition of corrosion. By reducing the concentration of these corrosive elements in the immediate vicinity of the metal surface, ACNPs effectively slow down the corrosion process.

The results of the dropping point test for the jojoba grease samples with and without ACNPs are shown in Table 2. Commercial lithium grease is also tested in this work for comparison. The thickener strongly identifies the grease rheological and physicochemical properties. The lithium soap has a typical spiral fibrous structure resulting in a high entanglement level, which leads to minimum oil separation and the highest dropping point. Although G1 sample shows a dropping point that is lower than that of lithium grease, the addition of ACNPs in the G2 and G3 samples increases the dropping point by up to 8%, which is acceptable for machinery applications, such as rolling bearings. Porous materials such as AC improve the colloid stability of grease by retaining part of the base oil when added to grease. This reduces the amount of oil separating from the grease at higher temperatures^73,74^. As such, the inclusion of ACNPs enhanced the grease's colloid stability, leading to an increase in dropping points, as observed.

### Jojoba grease tribological properties

**Figure 4:**
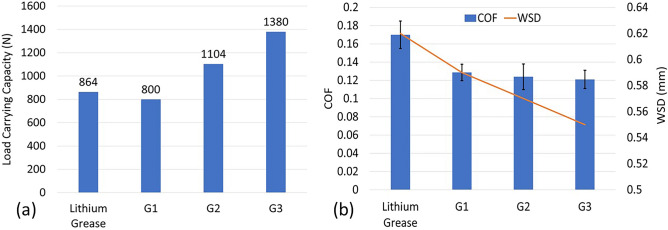
(**a**) Load carrying capacity and (**b**) WSD and COF of grease samples.

Figure 4a shows the results of the load carrying capacity for the jojoba grease samples. Commercial lithium grease is also tested for comparison. G1 sample shows the lowest load carrying capacity with only 800 N. Adding ACNPs to jojoba grease increases the load carrying capacity by 38% and 72% for G2 and G3 samples compared to G1 grease. The figure also shows that G2 and G3 samples (jojoba grease with ACNPs) exhibit higher load carrying capacity than lithium grease. This can be explained by the good mechanical properties of AC as a carbonaceous material, such as the modulus of elasticity and damping ratio^43^.

The WSD and COF results are shown in Fig. 4b. The average WSDs of G1, G2, and G3 samples are 0.59 mm, 0.57 mm and 0.55 mm, respectively. G1 sample exhibited a reduction in the WSD value by 20% when compared to lithium grease (0.73 mm). The addition of ACNPs to jojoba grease reduced the WSD by 22% and 25% in the case of G2 and G3 samples, respectively. Figure 4b shows the average COF values from three repeated friction tests for jojoba grease samples according to ASTM 5183. The results demonstrate that lithium grease has the highest COF of approximately 0.17. G1 sample, on the other hand, shows an average value of 0.149, which is 13% lower than that of lithium grease. This reduction in the friction coefficient can be attributed to the high VI for jojoba oil (242.1). It can also be noted that jojoba grease containing 0.5 wt.% and 1.5 wt.% ACNPs additives demonstrate lower COF values than lithium grease by 33% and 38%, respectively.

**Figure 5:**
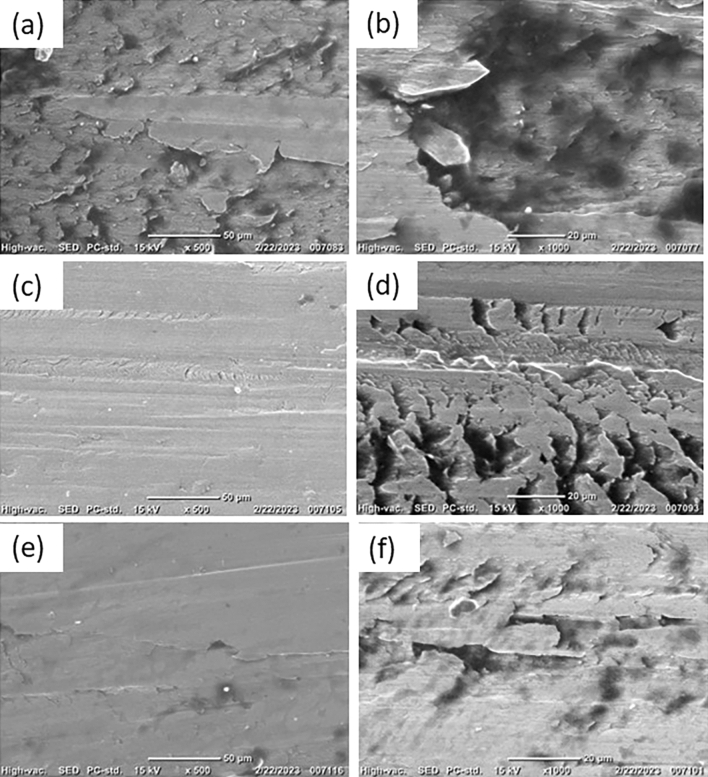
SEM images of test balls for: (**a**) G1 sample (50×), (**b**) G1 sample (20×), (**c**) G2 sample (50×), (**d**) G2 sample (20×), (**e**) G3 sample (50×), and (**f**) G3 sample (20×).

**Figure 6:**
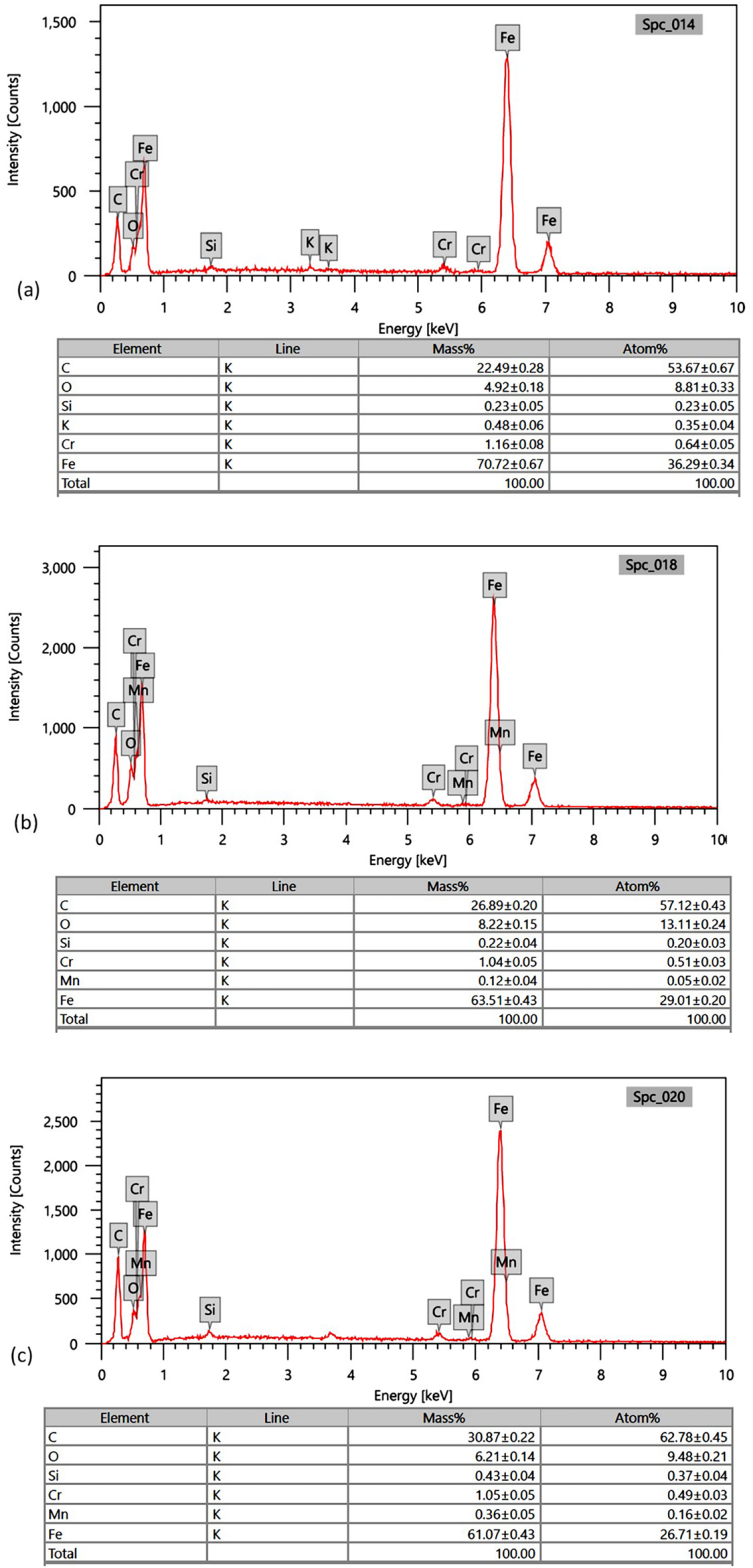
EDX spectra for SEM wear scars images at magnification 200× for: (**a**) G1, (**b)** G2, (**c**) G3 grease samples.

To further understand the friction and wear mechanisms under jojoba grease lubrication, SEM coupled with energy dispersive X-ray (EDX) analysis is applied on the wear scar area of the test balls from tribological tests. Figure 5 shows the worn surface morphology for the test balls at magnifications of 500× and 1000×. The balls lubricated with G1 grease sample (Fig. 5a and b) demonstrated a rough scar surface with severe scuffing. Friction forces at elevated temperatures during the test time cause noticeable surface layer delamination, which suggests a dominant adhesive wear as a primary wear mechanism. In the case of the G1 sample, the formation of the tribolayers between the rubbing contacts is predominantly attributed to the presence of long chains fatty acids in jojoba oil, along with a substantial amount of wax esters. When exposed to high-pressure conditions during friction, these esters are easily released from the thickener structure within the contact area and then adhere to the contacting surfaces. By observing the EDX results for G1 sample in Fig. 6, the presence of 22.49 ± 0.28 wt.% carbon (C) and 4.92 ± 0.18 wt.% oxygen (O) on the worn surface of the ball bearing element implies the existence of the jojoba fatty ester constituents as an adhered film on the rubbed steel surface, lowering friction and wear by the previously reported levels. However, it's important to note that the strength of the resulting tribofilm is quite limited, providing only minimal protection to the contacting bodies. This behavior undergoes a significant transformation when examining samples reinforced with ACNPs.

Looking at bio-grease samples of G2 and G3, shallow micro plows with fewer grooves with visible traces of the ACNPs are lodged on the surface are observed, as shown in Fig. 5c–f. This observation is proved by the EDX results in Fig. 6b where the worn surface in G2 sample possesses higher carbon content of 26.89 ± 0.20 wt.% C and 8.22 ± 0.15 wt.% O in comparison with G1 sample. It is believed that ACNPs tribofilm layer was strongly adsorbed to the surface causing a shift from adhesive wear to abrasive wear mechanism between rubbing surfaces. The least wear is observed in the G3 sample, where larger quantities of carbon (30.87 ± 0.22 wt.% C) were detected by EDX, indicating the formation of more stable tribofilm. Based on the AC microstructure results from SEM analysis, it is believed that ACNPs have the capability to fill the gaps between surface irregularities, resulting in smoother worn surfaces. Additionally, the C=H and C=C functional groups in ACNPs facilitate the formation of protective barriers on the metal surface, ensuring comprehensive coverage due to their aggregate structure and substantially large surface area, as indicated by Brunauer, Emmett and Teller (BET) test results (448.88 m^2^/g) in^42^.

### Bearing test results

**Figure 7:**
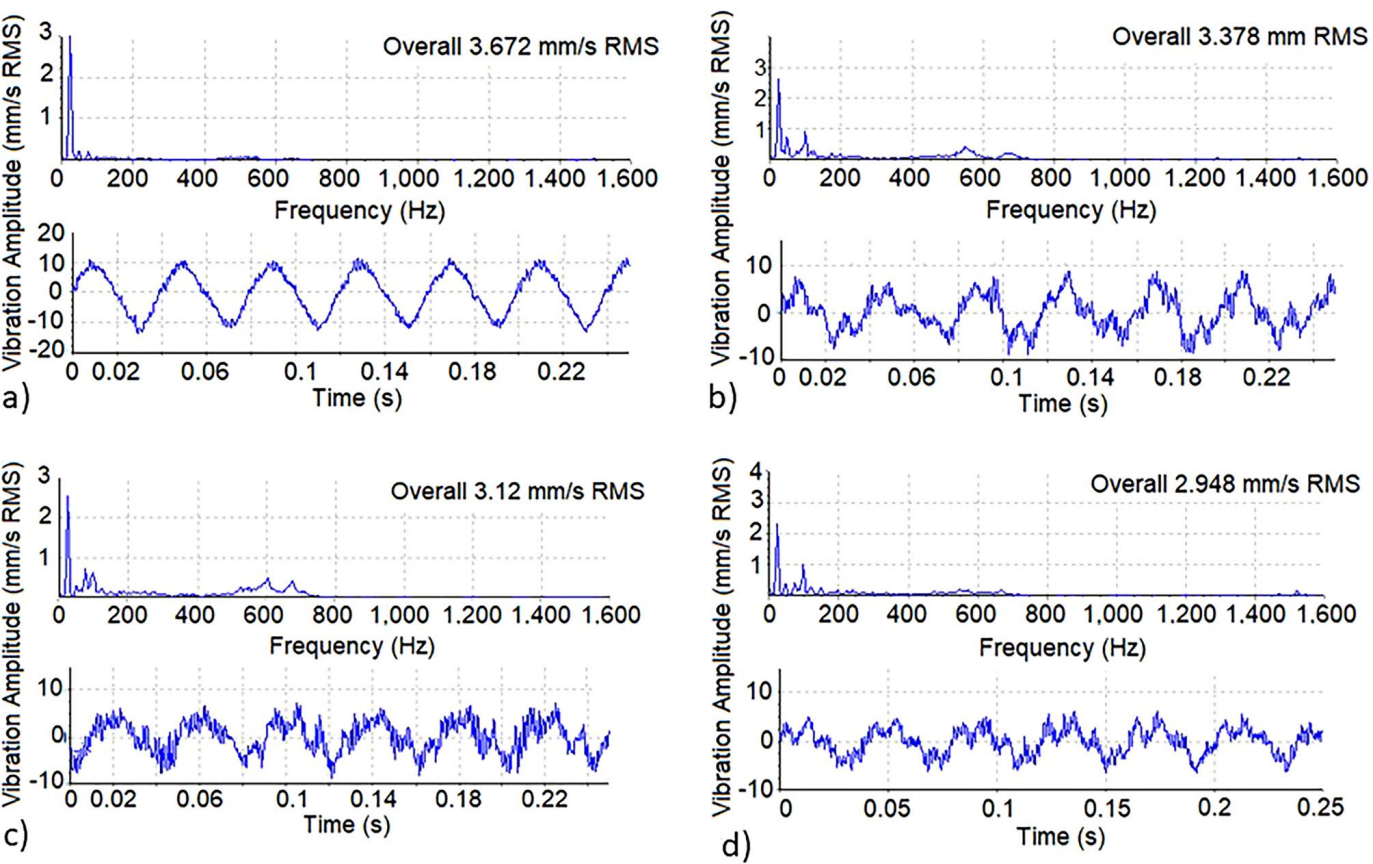
Time waveform and frequency spectrum of ball bearings loaded with 100 N deadweight and lubricated by: (**a**) commercial lithium grease, (**b**) G1 grease sample, (**c**) G2 grease sample, and (**d**) G3 grease sample.

**Figure 8:**
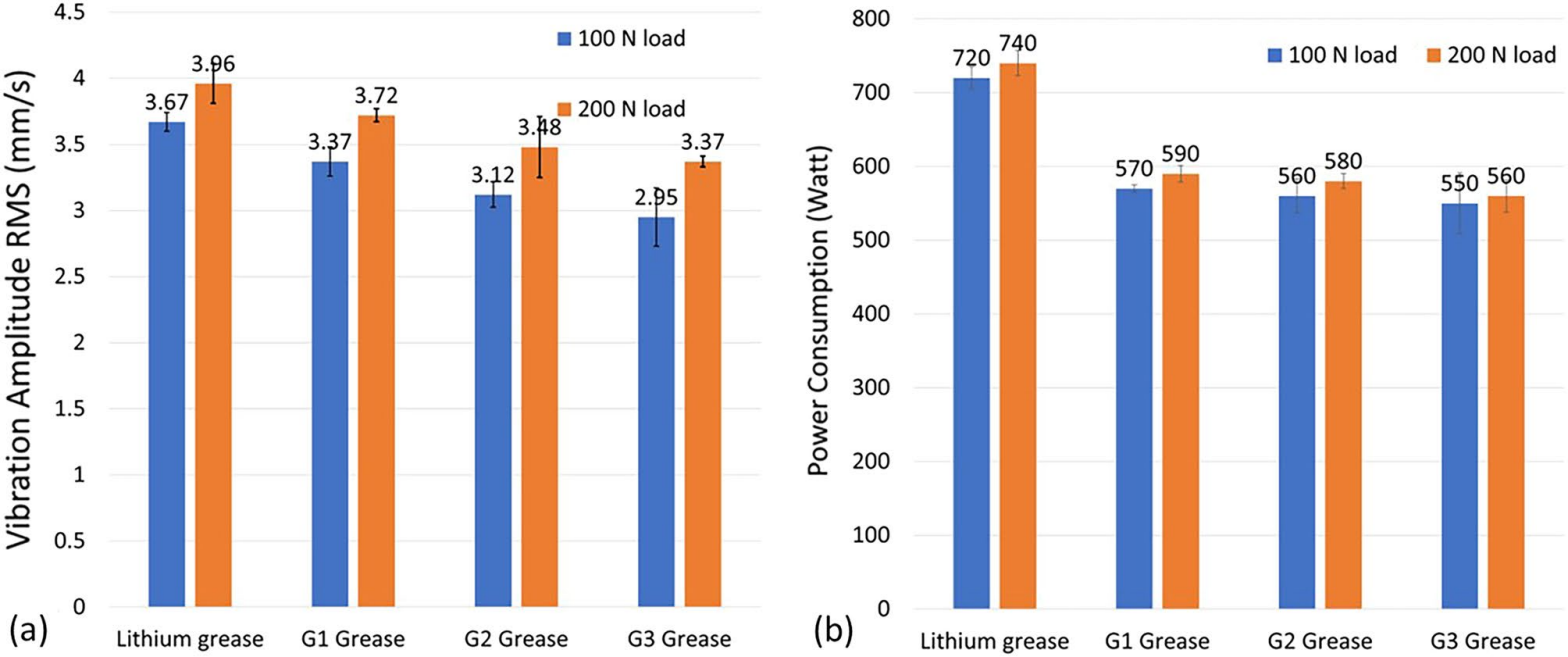
(**a**) Vibration levels (RMS, mm/s) measured in radial vertical direction, and (**b**) power consumption values during operation of each grease sample under 100 N and 200 N loads.

Figure 7 shows the vibration measurements in radial vertical direction for test bearings lubricated with the investigated grease samples. The summary of measured vibration values (RMS) for all test bearings is demonstrated in Fig. 8a.

During a 30 min running test, the vibration levels in case of rolling bearing lubricated with lithium grease reached 3.67 and 3.96 mm/s at radial loads of 100 N and 200 N, respectively. These levels are found below the maximum permissible limits according to ISO 10816^75^. Test bearings lubricated with G1 grease (without additives) show slightly lower vibration levels. The addition of ACNPs in G2 and G3 samples to rolling bearings resulted in considerable reductions in the vibration amplitude reaching 3.12 and 2.95 mm/s under 100 N radial load. Increasing the weight percentage of ACNPs in grease sample to 1.5 wt.% resulted in a reduction in vibration levels by 20%.

Vibration signal components corresponding to lubricant deficiencies appear at high frequency bands between 900 and 1600 Hz as sharp vibration peaks^76^. Insufficient viscosity grade will lead to a minimum oil film thickness which is unable to separate bearing mating surfaces. In consequence, severe rubbing action will take place between the mating surfaces in direct contact at the asperity level causing excitation of several resonant modes^77–79^. The ratio of oil film thickness to surface roughness of mating surfaces is known as λ parameter and it is used as an indicator of the lubrication regime whether it is elasto-hydrodynamic lubrication (EHL) full film or mixed film or boundary lubrication. In a previous work^77^, ISO 10 oil was tested in roller bearing under different applied loads and speeds. During the first hour of operation, the oil achieved a full film EHL between bearing components which was proved by λ parameter calculations and vibration levels. In this work, jojoba oil viscosity (without additives) is equivalent to ISO 22 which is higher in viscosity grade than ISO10 oil, hence, it is expected to provide a more stable oil film thickness that ensures operating in EHL full film zone for the tested ball bearing. As a consequence, no metal-to-metal contact takes place which is confirmed by the low and acceptable vibration RMS levels and by the absence of resonant peaks at high frequency zones in Fig. 7.

Jojoba oil is predominantly made up of liquid wax rather than triglycerides. Furthermore, wax esters are esters of mono-unsaturated long-chain fatty acids with high molecular weight monohydroxy alcohols. This is reflected on the calculated high VI (242.1). In G2 and G3 samples, further enhancement in kinematic viscosity and VI values of jojoba oil with the presence of ACNPs is believed to generate of a steadier tribofilm oil layer within the raceway of the bearing under the same applied loads and operating temperature. This explains the reduction in vibration levels for bearings lubricated with G2 and G3 grease samples^80^. Another possible reason is that carbonaceous nanoadditives exhibit high damping and elasticity properties that effectively decrease the transmitted vibrations between bearing elements^43^. It is believed that ACNPs existing in the formed tribofilm layer between the rolling elements and bearing raceways act as spring and damper connectors that reduce transmitted vibrations. Furthermore, the agglomerated and bulky shaped ACNPs with the average size of 37 nm covers the asperities of the mating surfaces with magnitudes of 0.5 µm and hence, they are supposed to lessen metal-to-metal contact chances along the contact surface.

Power consumption is measured to evaluate the energy losses during operation due to friction when using investigated grease samples for bearing lubrication. Figure 8b shows the consumed power levels using a wattmeter connected to the induction motor. Among all test samples, bearing lubricated with lithium grease results in the highest power consumption rates of 720 W and 740 W, respectively. Replacing lithium grease with jojoba grease G1 resulted in a reduction of 20% in power consumption. In the case of applying G2 and G3 greases, this enhancement reaches between 22 and 24% power saving. The results of power consumption confirm the results of COF and vibration measurements.

Overall, jojoba oil proved to be a competent lubricant with adequate tribological and phsyciochemical behavior when compared to some other bio-greases from review of literature. For example, jojoba grease with and without ACNPs showed COF values similar to findings of bio-grease samples from castor oil with calcium soap, castor oil with methylcellulose, and castor oil with chitin, and cellulosic pulp^81^. Furthermore, jojoba oil showed smaller WSD by 33% than castor oil with calcium soap and better wear scar morphology due to the presence of ACNPs.

## Conclusions

This paper presents a new grease lubricant comprised of jojoba oil and activated carbon nanoparticles (ACNPs) as additives extracted from banana peel waste. Jojoba grease samples are prepared in their plain state and with blends of 0.5 wt.% and 1.5 wt.% ACNPs. The samples are then examined for copper corrosion tendency, dropping point, COF, WSD, load carrying capacity, and the results are compared with commercial lithium grease. The dynamic performance of jojoba grease samples was also evaluated using 6006zz ball bearings running at different loads by measuring vibration levels and power consumption. The following conclusions are drawn:

- Characterization results of ACNPs revealed a high purity of structure, acceptable nanoparticle sizes proving the efficiency of producing ACNPs from banana peels as a reliable and sustainable source.
- The tests on raw jojoba oil manifested adequate physicochemical properties in terms of VI (242.1), copper corrosion rating (1a), low water content % (0.03), and acid value (0.2).
- The jojoba grease without ACNPs additives resulted in corrosion resistance of 2c due to the presence of stearic acid in the lithium soap and the absence of a corrosion inhibitor. The addition of ACNPs in the G2 and G3 samples enhanced the corrosion resistance rating to (1a) without degrading the stability of jojoba grease.
- The functional groups in ACNPs are specific chemical moieties attached to the surface of the nanoparticles, which can interact with the metal surface and the corrosive environment. These interactions enhance the corrosion inhibition properties of ACNPs. Consequently, the dropping point is enhanced from 100 to 109 degrees with the addition of ACNPs.
- The tribological results demonstrate that enhanced jojoba grease with ACNPs outperforms lithium grease. The load-carrying capacity of jojoba grease is increased by up to 60% when 1.5 wt.% ACNPs are added, while the COF and WSD are enhanced by up to 38% and 24%, respectively. The functional groups such as hydroxyl (–OH), carboxyl (–COOH), and amino (–NH) groups create a stable and robust passive tribofilm that significantly reduces the COF and WSD during the rubbing action between the mating surfaces.
- The addition of ACNPs caused a noticeable reduction in the bearing vibration RMS values and power consumption levels in comparison with lithium grease. This proves the generation of a stable minimum oil film thickness that ensures the bearing operation in EHL full film lubrication zone.

## Data and code availability

The data that support the findings of this manuscript are available from the corresponding author upon request.
